# Long-term neurological and healthcare burden of adults with Japanese encephalitis: A nationwide study 2000-2015

**DOI:** 10.1371/journal.pntd.0009703

**Published:** 2021-09-14

**Authors:** Hsuan-Ying Chen, Chen-Yi Yang, Cheng-Yang Hsieh, Chun-Yin Yeh, Chang-Chun Chen, Yen-Chin Chen, Chung-Chih Lai, Rebecca Claire Harris, Huang-Tz Ou, Nai-Ying Ko, Wen-Chien Ko

**Affiliations:** 1 Institute of Clinical Pharmacy and Pharmaceutical Sciences, College of Medicine, National Cheng Kung University, Tainan, Taiwan; 2 Department of Neurology, Tainan Sin Lau Hospital, Tainan, Taiwan; 3 Department of Computer Science and Information Engineering, College of Electrical Engineering and Computer Science, National Cheng Kung University, Tainan, Taiwan; 4 Department of Nursing, College of Medicine, National Cheng Kung University, Tainan, Taiwan; 5 Department of Nursing, National Cheng Kung University Hospital, College of Medicine, National Cheng Kung University, Tainan, Taiwan; 6 Global Medical Franchises, Sanofi Pasteur, Singapore; 7 Vaccine Epidemiology and Modelling, Sanofi Pasteur, Singapore; 8 Department of Pharmacy, College of Medicine, National Cheng Kung University, Tainan, Taiwan; 9 Department of Medicine, College of Medicine, National Cheng Kung University, Tainan, Taiwan; WRAIR, UNITED STATES

## Abstract

**Objective:**

To assess the healthcare utilization, economic burden, and long-term neurological complications and mortality of an adult population with Japanese encephalitis (JE).

**Methods:**

This study utilized two nationwide datasets in Taiwan: the Notifiable Disease Dataset of confirmed cases from the Centers for Disease Control to identify JE patients, and the National Health Insurance Research Database to obtain patients’ healthcare utilization. Survival analyses were performed to identify prognostic factors associated with the all-cause mortality of patients.

**Results:**

This study included 352 adult cases with JE (aged≥20 years). The mean age of JE patients was 45 years. Stroke (event rate: 3.49/100 person-years) was the most common neurological complication, followed by epilepsy/convulsions (3.13/100 person-years), encephalopathy/delirium (2.20/100 person-years), and parkinsonism (1.97/100 person-years). Among the 336 hospitalized patients at JE diagnosis, 58.33% required intensive care. Among 79 patients who died following JE diagnosis, 48.84% of death events occurred within the year of diagnosis. The medical costs increased considerably at JE diagnosis and subsequent-year costs remained significantly higher than the costs before diagnosis (*p*<0.05). Having a four-dose JE vaccination (i.e., born after 1976) versus no JE vaccination history (i.e., born before 1963) was significantly associated with lower all-cause mortality (hazard ratio: 0.221 [95% confidence interval: 0.067, 0.725]). Comorbid diabetes and incident epilepsy/convulsion events significantly increased the mortality risk by 2.47- and 1.85-fold, respectively (*p*<0.05).

**Conclusion:**

A considerable medical burden associated with JE was observed in affected adults, even in the years following JE diagnosis. Vaccination should be considered to prevent this sporadic, but lethal, viral infection.

## Author summary

The epidemiology of adulthood Japanese encephalitis (JE) remains limited, and data on the economic burden associated with JE is lacking. This study is the first to comprehensively examine the healthcare burden (i.e., healthcare utilization and costs, neurological complications, all-cause mortality) of an adult population with JE, utilizing a nationwide cohort of JE-infected adults with up to 16 years of follow-up. In the first 6 months following JE diagnosis, a higher rate of neurological disorders was found, compared to the years after the diagnosis, with stroke being the most common neurological complication, followed by epilepsy/convulsions. The healthcare utilization of JE patients was higher in the first 6 months after the diagnosis compared to the years following the diagnosis. Medical costs increased considerably at JE diagnosis and subsequent-year costs after diagnosis remained higher than the cost before diagnosis. Having comorbid diabetes or incident epilepsy/convulsion events was a significant risk factor for mortality of adults with JE. Being born after 1976 in Taiwan, and thus likely receiving a four-dose schedule of vaccination, was associated with reduced mortality. Therefore, special attention is required for JE patients with comorbid diabetes or incident epilepsy/convulsion events, and JE vaccination should be considered to prevent this sporadic but lethal viral infection.

## Introduction

Japanese encephalitis (JE), which is caused by the Japanese encephalitis virus, is a mosquito-borne disease commonly found in Asia and Australia [1,2]. In areas with endemic JE, the virus primarily affects children, with the vast majority of cases being children aged < 15 years [3–12]. As there is no effective antiviral treatment, vaccination is the only approach for preventing this virus [13]. In areas with childhood JE vaccination, although the overall incidence of JE has decreased, a greater proportion of adults affected by JE has been reported, predominantly in temperate Asian countries (e.g., Japan, China, South Korea) [14–22]. The phenomenon of increasing adult population with JE has also been reported from South Asia where the routine vaccination program for JE was not available [23,24]. The epidemiology of childhood JE has been extensively studied [3–12]; however, the original epidemiology research with an assessment of infection in adults remains limited [14–18,20–22,25]. These limited studies used a cross-sectional study design (i.e., analysis of one-time outbreak [14–16]), consisted of few participants (case reports or case series studies [14,22]), or considered a specific region in a country (e.g., northern [20] or southern [21] Taiwan, a few cities in China [18], Gorakhpur in India [25]), and the majority of them were based on data collected in the 1990s or earlier [14–18,20,25]. Moreover, evidence of the economic burden associated with JE and its sequelae is still lacking.

The Japanese encephalitis virus causes particularly severe neurological manifestations leading to considerable loss of disability-adjusted life years, compared to any other arthropod-borne virus [13]. It has been estimated that approximately 20%-30% of JE patients die and that 30%-50% of JE survivors develop neurological, cognitive, or behavioral sequelae [13]. However, the majority of studies on JE-related neurological complications analyzed pediatric cases with a limited sample size (ranging from 29 [26] to 180 [27]), with relatively short follow-up periods (<2 years [26,28]), or from only certain hospitals [26–29]. Only two studies, one each from South Korea [22] and Taiwan [21], have reported adult cases with JE-associated short-term neurological impairments. However, the generalizability of their results is unclear due to the selective and limited number of study patients (i.e., 17 cases aged >20 years [22]) from certain hospitals [21,22] and a short follow-up period (i.e., only at the time of hospitalization at JE diagnosis).

Studies with long-term follow-up and large sample sizes are crucial for providing evidence of healthcare utilization and economic burden related to JE, especially from neurological, cognitive, or behavioral sequelae, which typically occur in chronic courses. The present study, therefore, conducted a nationwide study for adult patients with confirmed JE with up to 16 years of follow-up to report the healthcare utilization, economic burden, and long-term neurological and mortality outcomes of JE-infected adults. Additionally, because the risk factors attributable to severe outcomes in a JE-infected population are not fully elucidated, we examined the prognostic factors associated with the overall survival of adults with JE.

## Methods

### Ethics statement

This study was approved by the Institutional Review Board (IRB) of National Cheng Kung University Hospital (B-ER-106-272). The informed consents to study subjects were waived by the IRB committee because patients in the databases utilized in this study were deidentified.

### Data source and linkages for study cohort

A retrospective database analysis was conducted using data from two nationwide datasets in Taiwan, namely the Notifiable Disease Dataset (NDD) of confirmed cases from the Taiwan Centers for Disease Control (CDC) [30] and the National Health Insurance Research Database (NHIRD) [31,32]. The NDD data contain confirmed JE cases in each calendar year since 1999. Patients who met the JE-associated clinical conditions with positive test results were confirmed as JE cases in the NDD data. JE has been a notifiable communicable disease in Taiwan since 1955, whereby physicians are required to report incident cases. Study subjects were confirmed JE cases reported by the CDC between 2000 and 2014, and aged 20 years or above. Only two flaviviruses, the JE virus and the dengue virus, are known to circulate in Taiwan. An E/M-specific capture immunoglobulin M (IgM) and IgG enzyme-linked immunosorbent assay (E/M-specific IgM/IgG enzyme-linked immunosorbent assay [ELISA]) for JE and dengue was developed by the Taiwan CDC in 1998. Antibodies against both JE and dengue are screened using hemagglutination inhibition (HI) or the E/M-specific IgM/ IgG ELISA. Samples that test positive in the E/M-specific IgM/IgG ELISA are subsequently tested using the dengue ELISA to exclude the probability of cross-reactivity [33]. A previous study with a long-term evaluation demonstrated that the E/M-specific IgM/IgG ELISA was highly sensitive and specific, and it can effectively differentiate JE virus infection from dengue virus infection [34]. The JE-confirmed cases were defined as clinical cases that met one of the following laboratory criteria: 1) an HI titer of the convalescent serum of ≥1:160, and at least a four-fold rise between the HI titers of convalescent and acute sera; 2) an HI titer from a single serum sample of ≥1:320; 3) IgM positive serum by the ELISA test, or the IgG of the paired serum exhibited a four-fold increase; 4) a sample that was positive by real-time polymerase chain reaction assay; or 5) a sample that was positive to indirect immuno-fluorescence antibody staining after isolating the virus [33].

The NHIRD comprises individual longitudinal medical records from almost 23 million beneficiaries in Taiwan’s National Health Insurance (NHI) program (> 99% of Taiwan’s population), including outpatient, inpatient, and emergency department visits, and pharmacy claims for prescriptions [32]. The individual-level data from the NDD and the NHIRD were linked by individual, encrypted, de-identified information from the Health and Welfare Data Science Center. To assess healthcare utilization following JE diagnosis, we excluded patients without any medical records in the NHIRD from the study cohort. Outlines of the study databases, data linking procedure, and study cohort identification are available in S1 Fig.

### Identification of study variables

#### Baseline demographic and clinical characteristics

Each patient was followed up from JE diagnosis to the end of 2015, or death, whichever came first. Patient demographics including age at JE diagnosis, gender and place of residence at city and county level (i.e., Taipei area [including Taipei City, New Taipei City, Yilan County, Lienchiang County, Keelung City, and Kinmen County], Northern Taiwan [including Taoyuan City, Hsinchu City, Hsinchu County, and Miaoli County], Central Taiwan [including Taichung City, Nantou County, and Changhua County], South Taiwan [including Tainan City, Chiayi County, Chiayi City, and Yunlin County], Kao-Ping area [including Kaohsiung City, Pingtung County, and Penghu County], and others [including Hualien County, Taitung City, Green Island, and Orchid Island]) [35] were retrieved from the NDD data. Neurological events of interest included fifteen individual components of neurological impairment, which were measured from one month before JE diagnosis to the end of study follow-up using the International Classification of Diseases, Ninth Revision, Clinical Modification (ICD-9 CM) disease codes based on the outpatient and inpatient records of the NHIRD. The occurrence of a neurological event was confirmed based on at least two outpatient visits or one hospitalization due to the neurological event. Other comorbidities, including hypertension, diabetes, and coronary heart disease were also identified using the ICD-9 CM disease codes based on the inpatient and outpatient records of the NHIRD, one year to one month before JE diagnosis. Details of operational definitions for study variables are provided in S1 Table.

In 1976 in Taiwan, the national compulsory immunization programme begun a four-dose schedule of a mouse-brain-derived JE vaccine, whereby all children below the age of 3 are required to receive two doses, with the third dose being administered one year later, and the fourth those being administered when the child is in their first year of elementary school. Owing to this immunization program, the vaccination coverage in Taiwan is generally high; >80% coverage for the two-dose and three-dose schedules, and >95% coverage for the four-dose schedule have been reported previously [17]. In the present study, as individual-level JE vaccination data were not available, JE vaccination exposure history was measured using birth cohorts as proxies, which were classified into four categories: i) likely no vaccination (born in the years without universal JE vaccination coverage; before 1963), ii) likely two doses of vaccination (born in 1963 to 1969), iii) likely three doses of vaccination (born in 1970–1975), and iv) likely four doses of vaccination (born after 1976).

#### Healthcare utilization

Healthcare utilization of study subjects in the year of, and years following JE diagnosis was obtained using the ICD-9 procedure codes, including intensive care unit (ICU) admission and length of stay, hospital admission and length of stay, airway suctioning, ventilation based on inpatient records of the NHIRD, nasogastric tube (NG)/percutaneous endoscopic gastrostomy (PEG) and urinary catheterization obtained from the emergency department, inpatient, and outpatient records. Details of ICD-9 procedure codes are presented in S2 Table.

#### Healthcare costs

Details about estimation for healthcare utilization and cost attributable to disease burden based on the NHIRD in this study are available in previous literature [36–39]. Specifically, the medical costs included in the NHIRD claims consist of fees associated with disease diagnosis, treatments (e.g., examinations, procedures, and special materials), pharmaceutical services, medications, and rehabilitation-related services. The economic analysis in this study was conducted based on the perspective of the healthcare sector. So, two aspects of medical costs in the formal healthcare sector were considered: i) direct medical costs paid by Taiwan’s NHI program (a third-party payer) and ii) out-of-pocket costs paid by individual beneficiaries (i.e., patients’ co-payments in this study). Cost values over 16 years of follow-up were standardized according to the medical care component of Taiwan’s consumer price index (https://eng.stat.gov.tw/public/data/dgbas03/bs3/english/cpiidx.xls) and then converted to 2019 US dollars using an average exchange rate of US$1:NT$30.905.

#### All-cause mortality

A patient’s mortality was confirmed from the cause of death file in the NHIRD [40]. The dis-enrollment records in the NHIRD registration files of beneficiaries were further used to confirm the mortality status [41].

### Statistical analyses

Means and standard deviations are used to present continuous variables, frequencies and percentages are applied to dichotomous variables (e.g., a specific healthcare service/procedure use) and categorical variables (e.g., residence area). Baseline characteristics were also stratified by the ICU admission (yes/no) at JE diagnosis and death/survival status following JE diagnosis. Differences in baseline characteristics between stratified subgroups were tested using the chi-square test. Based on the patients without any prior neurological disorders, the incidence rates of neurological events following JE diagnosis were estimated and stratified by time since the incident event occurred (the first 6 months, 7 months to 1 year, and 1 to 2 years following JE diagnosis).

The economic burden attributable to JE was estimated as event-year and annual state (subsequent)-year costs. Event-year costs were the costs associated with medical management of an acute care episode due to JE (initial management in emergency department, inpatient, or outpatient care setting) and any subsequent care provided within the first year of JE diagnosis. State-year costs reflected the annual resource use required beyond the first year for ongoing medical management while a given health state/chronic event (i.e., JE-related long-term health impairments) was present for the remainder of the study follow-up following JE diagnosis. Event and state costs over follow-up years were further stratified by inpatient, outpatient, emergency room, pharmacy, and rehabilitation-related costs. The Cochran-Armitage test for trend was carried out to assess the difference in the medical costs of JE patients over time, namely from the year before (baseline cost), in the year at (event-year cost), and the years following JE diagnosis (annual state-year costs).

A Kaplan-Meier survival curve was obtained to illustrate the overall survival of study patients after JE diagnosis over the study period (up to 16 years). Univariate Cox models were used to identify patient characteristics that were significantly associated with the overall survival of JE-infected adults. The characteristics considered were age at JE diagnosis (<40, 40–64, or ≥65 years), gender, residence area (Taipei, north, central, south, Kao-Ping, or other regions), JE diagnosis year (2000–2004, 2005–2009, or 2010–2014), prior disease status (comorbidities and neurological history), and JE vaccination history. Colinearity among the statistically significant variables obtained from the univariate analyses was examined using a variance inflation factor (VIF). A multivariate Cox model that included the variables producing a statistically and clinically significant impact on the patients’ survival was then conducted. All *p*-values were two-tailed, with values <0.05 considered as statistically significant. The statistical analyses were conducted using SAS 9.4 software (SAS Institute, Inc., Cary, NC, USA).

## Results

### Baseline characteristics

A total of 352 JE-infected adults were included in this study, representing approximately 20 cases per year in Taiwan during 2000–2014 (S1 Fig). S2 Fig illustrates the distribution of study adult cases with JE over the island of Taiwan. At JE diagnosis, 336 cases were admitted to hospital and 16 cases were only treated in outpatient care settings. There were 32 patients with prior neurological diseases (14 with stroke, 22 with neurological disorders, and 4 with both stroke and neurological disorders) in the year to 1 month before JE diagnosis; 320 patients did not have prior neurological events before JE diagnosis. The patient baseline characteristics (Table 1) were stratified by the status of ICU admission at diagnosis and all-cause death following the diagnosis. There were 236 cases with ICU admission records at JE diagnosis and 79 JE cases who died during the study observational period. More than half of the patients that were affected by JE were aged between 40–64 years (53.41%) and two-thirds of the cases were male (62.2%). Most individuals were from southern Taiwan (24.72% from south and 19.03% Kao-Ping regions) and born before the mass JE vaccination program (before 1963) was implemented (51%). Hypertension was the most prevalent comorbidity (21.31%) in our study patients followed by diabetes (9.94%).

**Table 1:** Characteristics of 352 study patients at JE diagnosis stratified by ICU admission status at diagnosis and survival status over study period after diagnosis.

Characteristics	N (%)^a^				
	Overall	With ICU admission (N = 236)	Without ICU admission (N = 116)	Survived (N = 273)	Died (N = 79)
**Age at JE diagnosis (years)**					
20–40	130 (36.93)	84 (35.59)	46 (39.66)	115 (42.12)	15 (18.99)*
40–64	188 (53.41)	125 (52.97)	63 (54.31)	143 (52.38)	45 (56.96)
≥65	34 (9.66)	27 (11.44)	7 (6.03)	15 (5.49)	19 (24.05)
**Gender**					
Female	133 (37.78)	88 (37.29)	45 (38.79)	104 (38.10)	29 (36.71)
Male	219 (62.22)	148 (62.71)	71 (61.21)	169 (61.90)	50 (63.29)
**Residence region at JE diagnosis**	0				
Taipei	45 (12.78)	32 (13.56)	13 (11.21)	31 (11.36)	14 (17.72)*
North	31 (8.81)	23 (9.75)	8 (6.90)	25 (9.16)	6 (7.59)
Central	82 (23.30)	46 (19.49)	36 (31.03)	73 (26.74)	9 (11.39)
South	87 (24.72)	61 (25.85)	26 (22.41)	66 (24.18)	21 (26.58)
Kao-Ping	67 (19.03)	46 (19.49)	21 (18.10)	53 (19.41)	14 (17.72)
Other	40 (11.36)	28 (11.86)	12 (10.34)	25 (9.16)	15 (18.99)
**Calendar year at JE diagnosis**					
2000–2004	109 (30.97)	70 (29.66)	39 (33.62)	81 (29.67)	28 (35.44)
2005–2009	128 (36.36)	93 (39.41)	35 (30.17)	96 (36.16)	32 (40.51)
2010–2014	115 (32.67)	73 (30.93)	42 (36.21)	96 (35.16)	19 (24.05)
**JE vaccine exposure history**					
Born before 1963: no vaccination	181 (51.42)	126 (53.39)	55 (47.41)	121 (44.32)	60 (75.95)*
Born in 1963–1969: 2 doses	68 (19.32)	42 (17.80)	26 (22.41)	60 (21.98)	8 (10.13)
Born in 1970–1975: 3 doses	48 (13.64)	30 (12.71)	18 (15.52)	40 (14.65)	8 (10.13)
Born after 1976: 4 doses	55 (15.63)	38 (16.10)	17 (14.66)	52 (19.05)	3 (3.80)
**Medical history at 1 year to 1 month before JE diagnosis**					
Comorbid hypertension	75 (21.31)	61 (25.85)	14 (12.07)*	44 (16.12)	31 (39.24)*
Comorbid diabetes	35 (9.94)	27 (11.44)	8 (6.90)	16 (5.86)	19 (24.05)*
Comorbid coronary heart disease	27 (7.67)	23 (9.75)	4 (3.45)*	14 (5.13)	13 (16.46)*
Stroke history	14 (3.98)	12 (5.08)	2 (1.72)	8 (2.93)	6 (7.59)
Neurological disease history^b^	22 (6.53)	18 (7.63)	4 (3.45)	13 (4.76)	9 (11.39)*
**CCI score**	0.94 (1.66)	1.11 (1.84)	0.59 (1.16)*	0.67 (1.45)	1.87 (1.99)*

Abbreviations: ICU: Intensive care unit, JE: Japanese encephalitis, CCI: Charlson comorbidity index.

Notes

^a^All variables are presented in N (%), excpect for CCI score (mean±SD).

^b^Neurological disease history included polyneuropathy/mononeuropathy multiplex and nerve root and plexus disorders.

*Indicates chi-squared test statistical difference (*p*<0.05) inpatient characteristics between ICU admission (yes vs. no) subgroups and between survived and died subgroups.

### Neurological complications at and following JE diagnosis

The incident neurological event rates over the study period after JE diagnosis among 320 patients who did not have prior neurological diseases in the year to 1 month before the diagnosis was obtained, as shown in Table 2. Epilepsy/convulsions were most frequent (15.93%) in the first 6 months after JE diagnosis. After the first year following JE diagnosis, stroke (6.25%) was the most frequently presented neurological disorder, followed by encephalopathy/delirium (5.86%), and polyneuropathy and mononeuropathy multiplex (5.86%). In the overall study period, stroke was the most common neurological disorder (event rate: 3.49/100 person-years), with epilepsy/convulsions as the second most common (event rate: 3.13/100 person-years).

**Table 2:** Incident neurological events and rates over the study follow-up period (up to 16 years after JE diagnosis) among 320 adult patients without prior neurological events in the year to 1 month before JE diagnosis^a^.

Neurological symptoms/signs	No. of incident events at the first 6 months of JE diagnosis, n (%)^b^ (N = 295)	No. of incident events at the first 7 months to 1 year of JE diagnosis, n (%) (N = 288)	No. of incident events more than 1 year after JE diagnosis, n (%) (N = 256)	Event rate over full study period (per 100 person-years) (N = 320)
Epilepsy/convulsions	47 (15.93)	2 (0.69)	9 (3.52)	3.13
Stroke	41 (13.90)	7 (2.43)	16 (6.25)	3.49
Parkinsonism	26 (8.81)	0 (0.00)	12 (4.69)	1.97
Encephalopathy/delirium	25 (8.47)	2 (0.69)	15 (5.86)	2.20
Polyneuropathy/mononeuropathy multiplex	12 (4.07)	3 (1.04)	15 (5.86)	1.52
Herpes zoster	6 (2.03)	2 (0.69)	12 (4.69)	0.96
Miscellaneous events^c^	18 (6.10)	1 (0.35)	17 (6.64)	1.89

Abbreviation: JE: Japanese encephalitis.

Notes

^a^Among a total of 352 study patients, there were 32 patients with prior neurological events (14 with stroke, 22 with neurological disorders, and 4 with both stroke and neurological disorders) in the year to 1 month before JE diagnosis.

^b^This column included the event that occurred in hospital when JE was diagnosed.

^c^Miscellaneous events included herpes simplex, dementia, facial nerve disorders, nerve root and plexus disorders, cerebral venous sinus thrombosis, and neuromuscular junction disorders; each of these events had an incidence rate (per 100 person-years) of less than 0.5.

### Healthcare utilization at and following JE diagnosis

Results of healthcare utilization at, and following, JE diagnosis is presented in Table 3. Among the 336 patients hospitalized at JE diagnosis, more than half of patients required admission to the ICU (196 cases; 58.33%), with an average length of ICU stay of 28.54 days. NG/PEG and urinary catheterization frequently occurred at JE diagnosis, and even in the years following diagnosis (average per person for NG/PEG and urinary catheterization at JE diagnosis versus in the years following diagnosis: 0.63 and 0.66 versus 3.78 and 1.62).

**Table 3:** Healthcare utilization at JE diagnosis to the end of study follow-up among all study patients (N = 352).

Features^a^	At date of JE diagnosis^b^	From date of JE diagnosis to first 6 months after diagnosis^b^	From first 7 months to 1 year after diagnosis^b^	From 1 year after JE diagnosis to end of follow-up^b^
No. of ICU admission (average per person)^c^	196 (0.56)	253 (0.72)	11 (0.03)	96 (0.27)
Mean length (days) of stat per ICU admission (Q1-Q3)	28.54 (14–38.5)	34.44 (16–43)	11.90 (5–14)	43 (13–57)
No. of hospitalizations (average per person)	336 (0.95)	542 (1.54)	77 (0.22)	551 (1.57)
Mean length (days) of stay per hospitalization (Q1-Q3)	26.59 (9.5–30.5)	52.95 (11–49)	62.82 (7–38)	103.14 (5–69)
No. of airway suctioning procedures (average per person)^c^	123 (0.35)	73 (0.21)	24 (0.07)	170 (0.48)
No. of ventilator uses (average per person)^c^	125 (0.36)	102 (0.29)	30 (0.09)	132 (0.38)
No. of NG or PEG procedures (average per person)	223 (0.63)	305 (0.87)	154 (0.44)	1,329 (3.78)
No. of urinary catheterizations (average per person)	232 (0.66)	248 (0.70)	103 (0.29)	569 (1.62)

Abbreviations: JE: Japanese encephalitis, ICU: Intensive care unit. NG: nasogastric tube; PEG: percutaneous endoscopic gastrostomy.

Notes

^a^A person could have several healthcare utilizations such as using a ventilator and nasogastric intubation at the same time during the observational period.

^b^Each of these study periods were mutually exclusive time intervals.

^c^ICU stay, airway suctioning, and ventilator use were defined in only the inpatient files of the National Health Insurance Research Database, whereas nasogastric intubation and urinary catheterization were defined in both inpatient and outpatient files.

### Medical costs before, at, and following JE diagnosis

**Fig 1:**
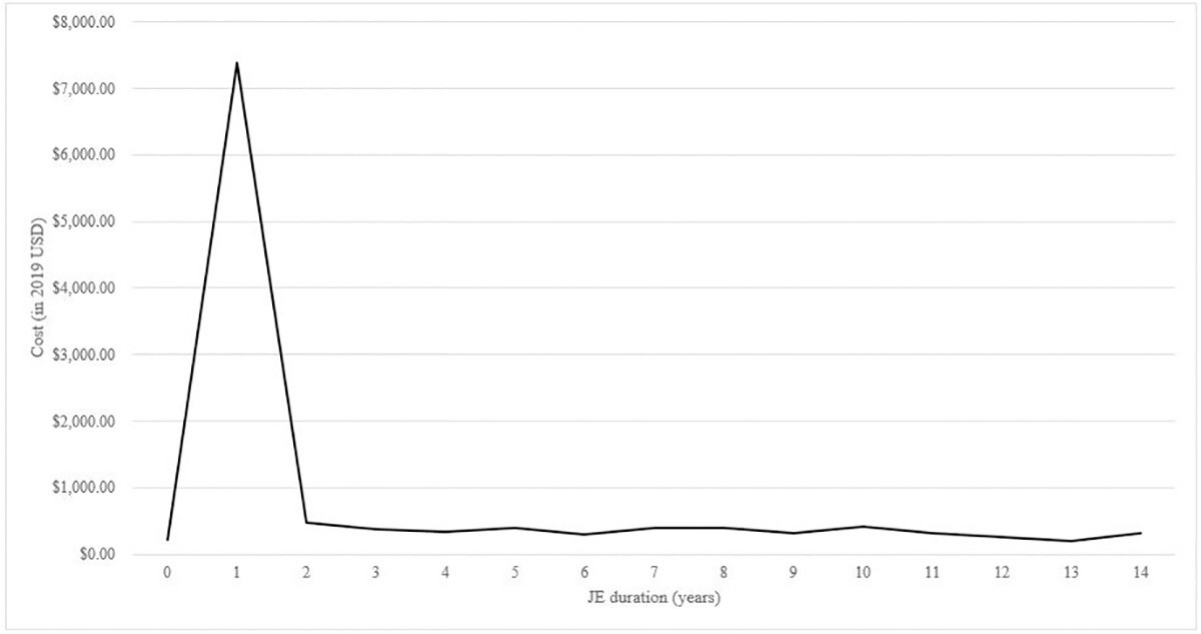
Crude annual healthcare median costs (in 2019 USD) of 352 JE-infected adult patients. Year 0: the year before Japanese encephalitis (JE) diagnosis (baseline year), Year 1: the year of JE diagnosis (event-year), Years 2–12: subsequent years after JE diagnosis (state-years).

The annual crude healthcare median costs in the year before (baseline cost), at (event-year cost), and following JE diagnosis (annual state/subsequent-year costs) are shown in Fig 1. There is a trend of medical costs increasing considerably at JE diagnosis. The median cost at baseline of $223.26 increased to a median event-year cost of $7,376.55 (*p*<0.05) and then decreased in the years after JE diagnosis to $350.79 (the median annual cost across Years 2–15), which remained higher than the baseline cost ($350.79 versus $223.26, *p*<0.05). Details of the cost breakdown are provided in S3 Table. The inpatient services accounted for most medical costs in the year at JE diagnosis (59.86%). The annual crude median healthcare costs stratified by the presence of comorbid diabetes and that of neurological complications are illustrated in S3 Fig. JE patients with comorbid diabetes or neurological events had higher medical costs compared to those without these diseases.

### Overall survival and associated prognostic factors among JE-infected adults

The Kaplan-Meier survival curve for overall survival of JE patients over the study follow-up period as shown in S4 Fig. Among 79 cases who died following JE diagnosis, 48.84% of deaths occurred in the year of diagnosis.

The results of univariate and multivariate Cox proportional hazards model analyses for all-cause death among 320 patients without prior neurological diseases are shown in S4 Table and Table 4, respectively. According to the results of the univariate analysis (S4 Table), age at JE diagnosis, likely JE vaccination history, baseline medical history (i.e., diabetes, hypertension, coronary heart disease), and incident events of epilepsy/convulsions were significantly associated with all-cause mortality. Considerable collinearity between age at JE diagnosis and grouped year of birth (as proxy for JE vaccination history) was found (VIF >10), only grouped year of birth was kept in the multivariate analysis because it was representative of both age and likely JE vaccination status. The final multivariate Cox model (Table 4) identified only three variables that were statistically associated with all-cause mortality. Patients who were born after 1976, and thus assumed to have had four doses of JE vaccination, had a significantly lower all-cause mortality versus those born before 1963, and thus assumed not to have been vaccinated (hazard ratio: 0.221; 95% confidence interval: 0.067, 0.725). In addition, patients with comorbid diabetes and those with incident epilepsy/convulsion events had a significantly increased all-cause mortality (2.47- and 1.85-fold, respectively) over the study time frame, compared to that of those without these comorbidities or incident neurological events.

**Table 4:** Results of multivariate Cox regression model for all-cause death as study outcome (based on 320 patients without prior neurological diseases).

	Hazard ratio	95% lower confidence limit	95% upper confidence limit
**JE vaccination history (ref: born before 1963: no vaccination)**			
Born in 1963–1969: 2 doses	0.491	0.213	1.132
Born in 1970–1975: 3 doses	0.668	0.290	1.538
Born after 1976: 4 doses	0.214	0.064	0.712
**Medical history at baseline (ref: none)**			
Comorbid diabetes	2.468	1.359	4.483
Comorbid hypertension	1.670	0.946	2.949
Comorbid coronary heart disease	1.389	0.626	3.078
**Incident events (ref: none)**			
Epilepsy/convulsions	1.845	1.061	3.209

Abbreviation: JE: Japanese encephalitis.

## Discussion

This is the first study to comprehensively examine the healthcare burden of an adult population with JE, including healthcare utilization and costs, long-term neurological complications, and all-cause death, utilizing a nationwide cohort of JE-infected adults with up to 16 years of follow-up. The present study therefore extends current knowledge about the healthcare burden attributable to JE in adult populations.

### Neurological complications following JE diagnosis

It has been previously shown that about half of JE survivors suffer from neurological or other sequelae at JE diagnosis and even in the years after the diagnosis, including various manifestations of neurological signs and symptoms (e.g., seizures, upper and lower motor neuron weakness, cognitive deficits, language impairment, psychiatric issues, learning difficulties, and behavioral problems). Most of this evidence was reported from pediatric studies. However, current data for JE-infected affected adults comes from very few studies [21,22] with selective and limited numbers of cases (i.e., certain hospital-based case reports or series) and only short-term clinical signs/symptoms of neurological outcomes (e.g., altered consciousness, fever, headache) being reported due to a limited follow-up time (e.g., the time of the first hospitalization at JE diagnosis).

Against this background, the present study is the first to assess relatively long-term neurological outcomes that could develop in the years after JE diagnosis. Based on a nationwide cohort of JE-infected adults with up to 16 years of follow-up, we found that most neurological symptoms or signs were observed in the first 6 months of JE diagnosis but could also occur years after the diagnosis (Table 2). Epilepsy/convulsions were the most frequent neurological complication in adults at JE diagnosis, followed by stroke, parkinsonism and encephalopathy/delirium. Pediatric studies have shown that seizures are common among pediatric patients [13] and associated with poor outcomes (e.g., mortality) in JE cases [42,43]. As a distinctive clinical presentation of JE, parkinsonism resulting from extrapyramidal involvement, with mask-like facies, tremor, cogwheel rigidity, and choreoathetoid movements reported in child JE cases [42,43]. Encephalopathy/delirium are also frequently present in childhood JE cases [44]. Additionally, in the present study of adult JE cases, stroke was among the most common neurological disorders after JE diagnosis over the study observation period. As reported in extreme cases, fever, headache, seizures, and changes in behavior or confusion could cause brain damage or even stroke [45,46]. This study, therefore, provides supportive evidence to highlight clinical importance of the early prevention, close follow-up for detection, and intervention of long-term neurological deficits in JE-infected adults following JE diagnosis.

### All-cause mortality among JE adults

JE populations generally have a fatality ratio of 20% to 30%, with some deaths occurring after a short fulminant course and others after a prolonged coma. In this study, 79 cases (out of 352 study patients, 22%) died during the study observation period, with 48.84% of death events occurring in the year of JE diagnosis (37 patients, S4 Fig). Previously, prognostic factors for mortality in JE-infected populations were poorly understood, therefore the present study explored the risk factors for all-cause mortality. Comorbid diabetes, incident epilepsy/convulsions, and JE vaccination history were significant prognostic factors in our analyses and deserve clinical attention. It has been reported that diabetes can worsen the condition of JE-infected patients by increasing neurological complications, which may happen due to facilitation of the virus movement across the blood-brain barrier [47], and possibly the alteration of plasma glucose levels, thus increasing the risk of hypoglycemia and consequently mortality [48]. Moreover, seizures, raised intracranial pressure (ICP) and brainstem signs which are commonly seen among JE patients have been strongly related to poor prognostic outcomes (e.g., mortality) of patients although the evidence is mostly from studies of pediatric populations. Uncontrolled seizures are related to various biochemical and metabolic consequences (e.g., hypoxemia, hypoglycemia, hyperlactatemia, low cerebrospinal fluid [CSF] glucose, high CSF lactate, metabolic acidosis, and CO_2_ retention) [49,50], leading to cerebral edema and a further rise in ICP, and thus a high mortality of patients [51]. Repeated seizures and status epilepticus were shown as strong prognostic indicators for death among children with JE [51]. Hence, interventions aimed at controlling these treatable comorbidities or neurological complications associated with JE may improve the survival outcome of patients. This study, therefore, highlights the importance of special care for the JE-infected patients who have diabetes or develop neurological complications after JE diagnosis.

Moreover, we found that being born in the years of universal coverage for the four-dose, but not two or three-dose, schedule of vaccination was associated with a significantly reduced all-cause mortality following JE diagnosis, when compared to those born before universal vaccination was introduced. Although JE is the most common vaccine-preventable cause of viral encephalitis and previous studies have shown that the mouse-brain-derived JE vaccine provides 96.8% protection after at least two doses, the protective antibody could be detected in only 32% of the patients in the three years after the final injection [52,53]. Other studies also have confirmed that the positive antibody rates decreased with time [33,52]. Therefore, a booster program with a four-dose schedule of the mouse-brain-derived JE vaccine may provide greater efficacy compared to that obtained with the two or three-dose schedule [54].

### Burden of JE in adulthood: healthcare utilization and costs

Despite most previous studies on JE patients reporting a high chance of ICU admission at the critical stage of JE diagnosis, the healthcare burden associated with JE in terms of healthcare utilization and costs has not been comprehensively assessed. This study found that the healthcare utilization of JE patients was higher in the first 6 months after diagnosis compared to the years following the diagnosis. In addition to over half of JE-infected adults having ICU admission at JE diagnosis, NG or PEG and urinary catheterization were also frequently present in the first 6 months after the diagnosis and these procedures were still needed among these adult patients in the years following the diagnosis.

Regarding the economic burden, the medical costs of JE patients significantly increased at the year of diagnosis (i.e., event-year cost) compared to the costs before diagnosis, and inpatient costs dominated the total healthcare costs of JE patients. Although the medical costs decreased in the years after JE diagnosis, they did not return to the baseline level in the ten years following the diagnosis; the subsequent-year costs after JE diagnosis remained higher than the costs before the diagnosis. Therefore, this study reveals the considerable economic burden of JE to the healthcare system at JE diagnosis and the substantial cost burden that may remain in the years after the diagnosis. Such an economic burden should be taken into consideration when countries decide immunization strategies and associated immunization coverage policies against JE. The results of this study could contribute to better-informed health economic analyses of JE immunization strategies.

### Study strengths and limitations

Compared to existing literature that either focuses on childhood JE or analyzes JE-infected adults with limited or selective cases and in short-term follow-ups, this study has several strengths. First, we targeted JE-infected adults, as confirmed by Taiwan’s CDC data. These patients had a relatively long follow-up time (up to 16 years), which allowed us to measure chronic neurological events (e.g., parkinsonism). Second, by using the individual-level data linked to the NHI claims data that cover all medical utilization and costs, this study is the first to report on the economic burden of adults with JE. The longitudinal nature of the data allowed us to explore the costs at the year of JE diagnosis as well as in subsequent years following the diagnosis among adult JE survivors. The NHI claims data are recognized as the most suitable source of costing information because of the large sample size, wide coverage (99%), detailed longitudinal cost data, and elimination of self-reported bias (i.e., recall and social desirability biases). Third, we analyzed incident neurological complications based on a subset of patients without any history of neurological complications before JE diagnosis (n = 320), unlike previous studies [21,22], which did not distinguish between prevalent and incident complication events. One could argue that analyses based on a complication-free cohort would provide more reliable and valid estimates of complication events that can be attributed to JE itself. Lastly, the prognostic factors for all-cause mortality were analyzed using multivariate model analyses that adjusted for possible risk factors to ensure the validity of study results.

Several limitations should be acknowledged. First, we utilized health claims data as the primary data source, where clinical biomarker, image and clinical sign/symptom data are typically lacking, which might affect the identification of neurological complications of JE. Also, some JE virus-infected patients may only present mild symptoms (e.g., fever, headache) and are not regarded as JE cases. So, they might not require medical care (e.g., hospital admission). Since most of our study JE patients had inpatient care at JE diagnosis, those with mild symptoms/signs of JE virus infection may be under-represented in our study cohort. Also, less severe complications for which patients did not seek medical care were likely to be missed; this might affect the estimates of healthcare utilization and cost burden associated with JE in the present study. Second, we could not differentiate if a stroke event is attributable to patients’ underlying comorbidities (e.g., diabetes, hypertension) or JE. This is warranted for future research to corroborate. Third, the present study did not consider the frequency or severity of neurological sequelae (e.g., a patient may have multiple episodes of a certain neurological sequelae; and, the number of episodes might imply the severity of the neurological disease) or model the disease severity in the survival analyses; this may underestimate the potential impact of neurological sequelae on patients’ survival outcomes. Also, considering a long-term follow-up for study patients (i.e., up to 16 years), the interpretation of the crude event rates of neurological events in the present study should be made with caution as these results might be affected by time-varying confounders over such a long period of time. Fourth, our cost analyses were conducted from the healthcare sector perspective in which we only considered the medical costs paid by a third-party payer (i.e., Taiwan’s NHI) and the co-payments made by patients (out-of-pocket costs). However, patients’ self-paid medical care/services were not analyzed because these costs were not available in the claims data. Fifth, because the details of JE vaccination of the laboratory-confirmed cases were not available and we used the birth cohorts as surrogate indicators for JE vaccination history, the vaccination history may have been overestimated. This problem might not be significant because the vaccination coverage rate in Taiwan has been generally high (i.e., coverage of >80% for two- and three-dose schedules and 95% for four-dose schedule [17]). However, as the exact vaccination record of individual study patients was unavailable, it is hard to clearly determine whether our results are age- or vaccination-history-associated effects. Further research, therefore, is needed to explore this potential association. Given the high vaccination coverage rate in Taiwan, the recommendation for clear documentation of JE vaccination history for individuals in national health databases should be made. As a result, clinicians could be aware of JE vaccination history for patients at JE diagnosis, which might have an impact on the long-term prognosis of patients. Lastly, the generalizability of the results in the present study might be limited to healthcare systems with universal health insurance coverage and a national compulsory immunization program for JE.

In conclusion, based on a nationwide cohort of confirmed JE-infected adults, a considerable burden associated with JE, in terms of healthcare utilization (e.g., ICU admission, NG/PEG use) and costs, neurological complications (e.g., stroke, epilepsy/convulsions) and mortality, was not only observed in the acute phase of JE diagnosis (i.e., the first 6 months of JE diagnosis) but also remained in the years after the diagnosis. In routine clinical care, special attention and medical care are required for JE patients with comorbid diabetes, and those developing epilepsy/convulsions after JE diagnosis, to reduce their mortality. Since JE could be a public health threat for adults, it is crucial for the people living in JE endemic areas to receive complete JE immunization. In particular, catch-up JE vaccination should be provided to adults who have not received the complete series of JE vaccination doses or have an unknown vaccination history. Therefore, from the perspective of public health policy, both routine JE immunization in childhood and catch-up JE vaccination for adult populations living in JE endemic areas should be available or even reimbursed through a national health insurance program.

## Supporting information

S1 Fig Flow chart of selection of JE patients confirmed by Taiwan’s CDC and linked with patient medical records in the NHIRD. Note: 79 cases confirmed from the CDC file died during the study period (i.e., 2000–2015). Abbreviations: CDC: Taiwan Centers for Disease Control, JE: Japanese encephalitis, NHIRD: National Health Insurance Research Database.
(TIFF)

S2 Fig **Distribution of study adult cases with JE over the island of Taiwan: (a) JE cases across the areas of Taiwan, (b) JE cases across the counties/cities of Taiwan**. Note: all the maps were created from Tableau Public (https://public.tableau.com/zh-tw/s/), and the base layer of the map was provided from Tableau Public.
(TIFF)

S3 Fig Crude annual healthcare median costs and those stratified by diabetes (DM) and neurological incident events (in 2019 USD) of 352 JE-infected adult patients. Year 0: the year before Japanese encephalitis (JE) diagnosis (baseline year), Year 1: the year of JE diagnosis (event-year), Years 2–12: subsequent years after JE diagnosis (state-years).
(TIFF)

S4 Fig Kaplan-Meier curve for all-cause death among a total of 352 JE-infected adult patients with up to 16 years of follow-up. (TIFF)

S1 Table ICD-9-CM disease codes for neurological events and comorbidities of interest in the present study. Abbreviation: ICD-9-CM: The International Classification of Diseases, Ninth Revision, Clinical Modification
(DOCX)

S2 Table ICD procedure codes for the healthcare utilization measured in the present study. Abbreviations: ICD: International Statistical Classification of Diseases, ICU: intensive care unit, NG: nasogastric tube; PEG: percutaneous endoscopic gastrostomy.
(DOCX)

S3 Table Annual crude healthcare cost breakdown for baseline cost before JE diagnosis, event-year cost at JE diagnosis and annual state/subsequent-year cost after JE diagnosis, and stratified by different medical services (i.e., inpatient, outpatient, emergency room, pharmacy and rehabilitation-related) among a total of 352 study patients (in 2019 USD). Abbreviations: s.d.: standard deviation, JE: Japanese encephalitis, ER: emergency room. Note: due to small values from the limited sample size at and after the 15^th^ year of JE diagnosis, the cost estimates are not presented in the table.
(DOCX)

S4 Table Results of univariate Cox proportional hazards model for all-cause death as study outcome. Abbreviation: JE: Japanese encephalitis. ^a^Neurological disease history included polyneuropathy/mononeuropathy multiplex and nerve root and plexus disorders.
(DOCX)
