# Supplementary figures and images for: Long-term neurological and healthcare burden of adults with Japanese encephalitis: A nationwide study 2000-2015
Source: PLoS Negl Trop Dis. 2021 Sep 14;15(9):e0009703. doi: 10.1371/journal.pntd.0009703 (PMC8486099; doi:10.1371/journal.pntd.0009703)

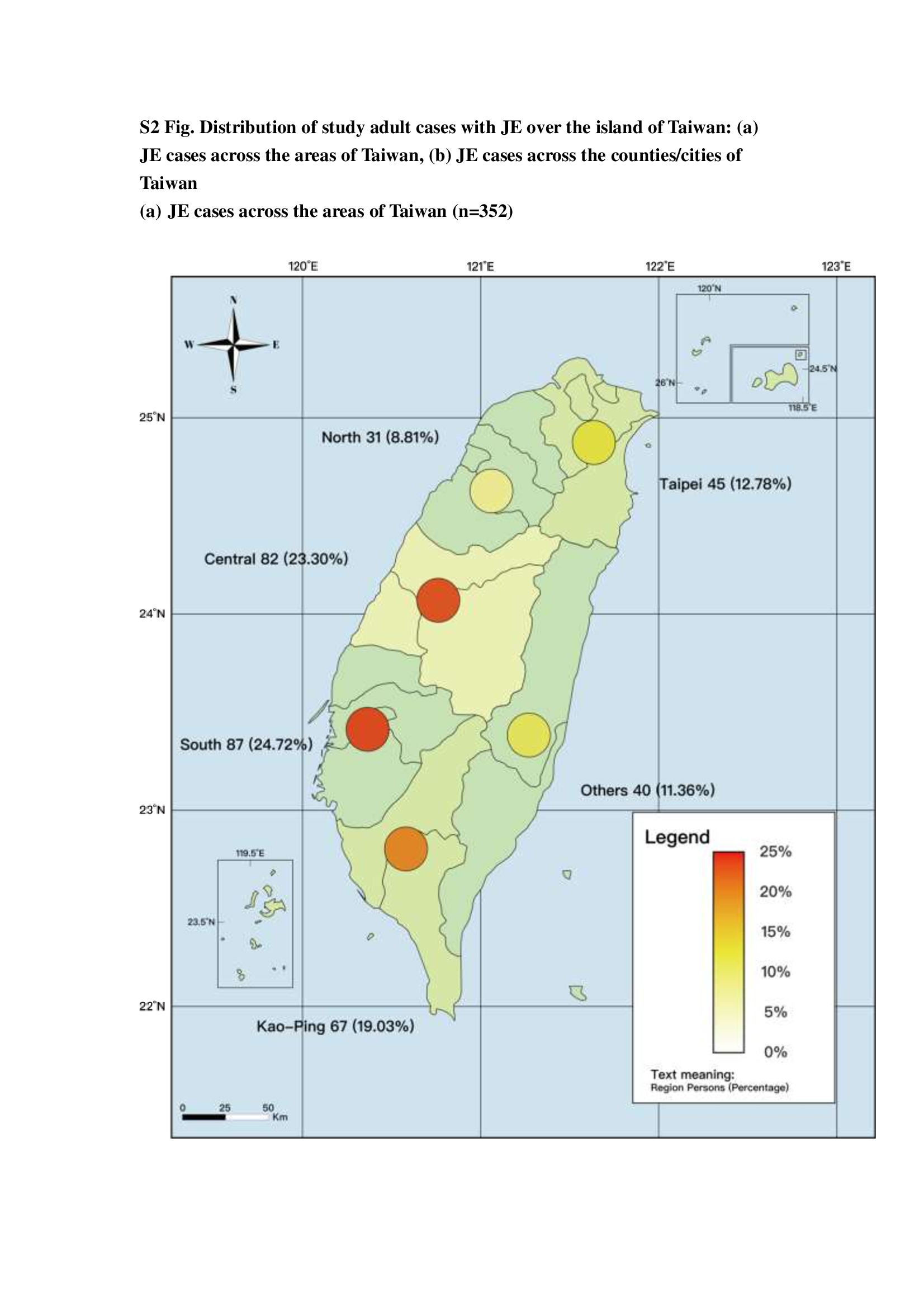

Supplement: S2 Fig — Distribution of study adult cases with JE over the island of Taiwan: (a) JE cases across the areas of Taiwan, (b) JE cases across the counties/cities of Taiwan. Note: all the maps were created from Tableau Public (https://public.tableau.com/zh-tw/s/), and the base layer of the map was provided from Tableau Public. (TIFF) [file pntd.0009703.s002.tiff]

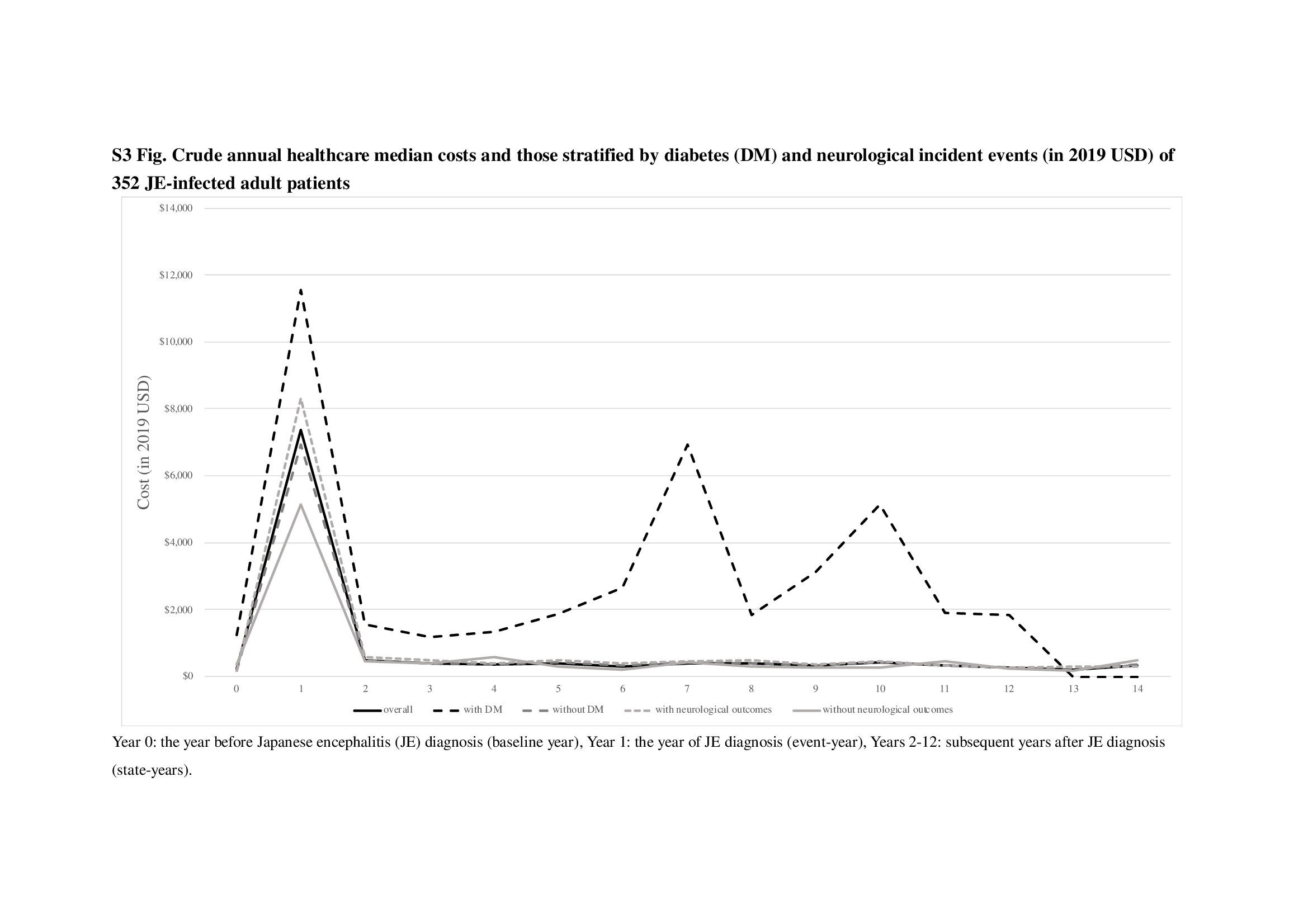

Supplement: S3 Fig — Year 0: the year before Japanese encephalitis (JE) diagnosis (baseline year), Year 1: the year of JE diagnosis (event-year), Years 2–12: subsequent years after JE diagnosis (state-years). (TIFF) [file pntd.0009703.s003.tiff]

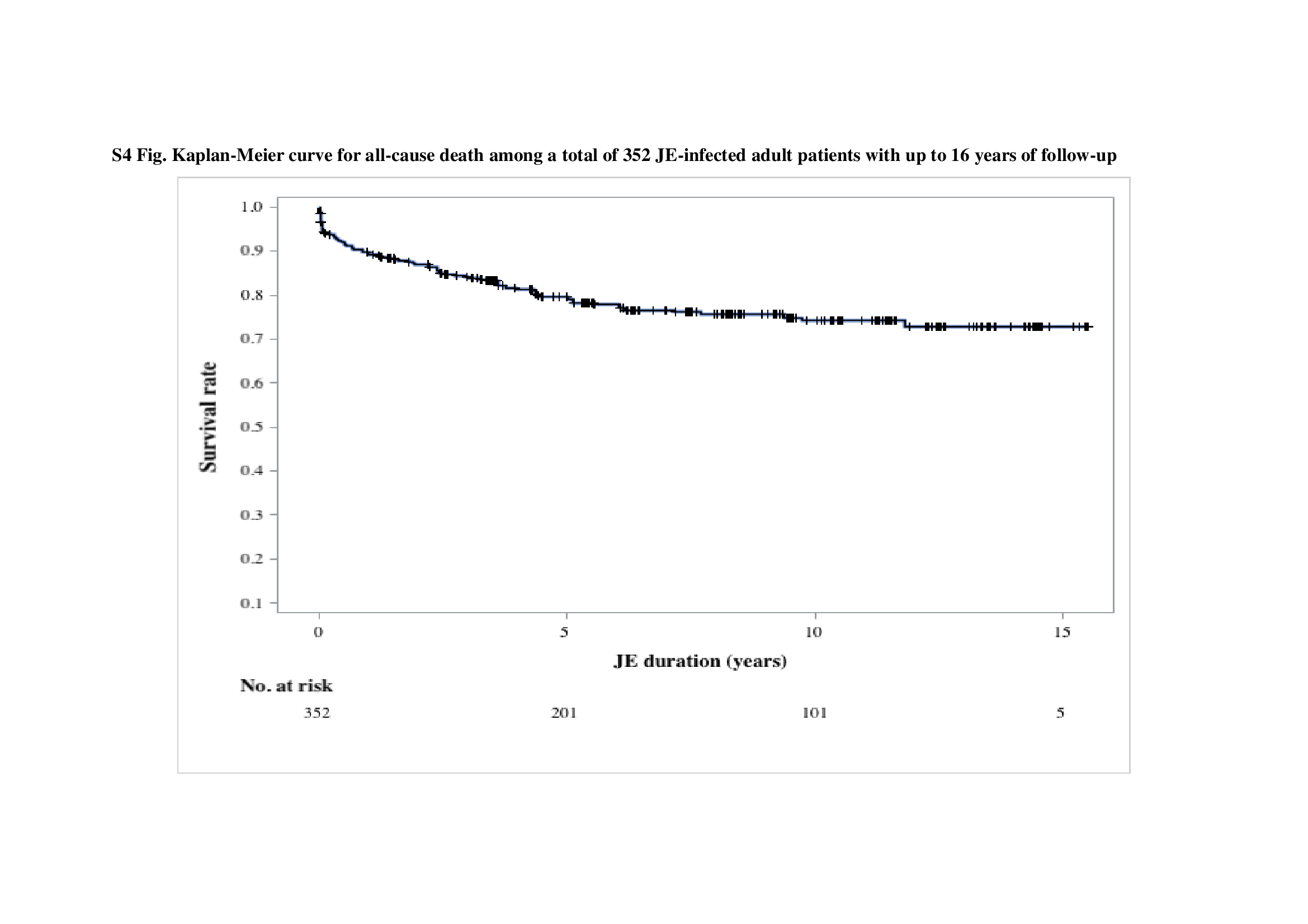

Supplement: S4 Fig — (TIFF) [file pntd.0009703.s004.tiff]
