# Supplementary material for: Long-term neurological and healthcare burden of adults with Japanese encephalitis: A nationwide study 2000-2015
Source: PLoS Negl Trop Dis. 2021 Sep 14;15(9):e0009703. doi: 10.1371/journal.pntd.0009703 (PMC8486099; doi:10.1371/journal.pntd.0009703)
Supplement: S1 Table — Abbreviation: ICD-9-CM: The International Classification of Diseases, Ninth Revision, Clinical Modification (DOCX) [file pntd.0009703.s005.docx]

**S1 Table. ICD-9-CM disease codes for neurological events and comorbidities of interest in the present study**

| Condition | ICD-9-CM Code | Description |
| --- | --- | --- |
| Epilepsy [1, 2] | 345 | Epilepsy and recurrent seizures |
| Convulsions [3] | 780.3 | Convulsions |
| Stroke [2] | 430 | Subarachnoid hemorrhage |
|  | 431 | Intracerebral hemorrhage |
|  | 432 | Other and unspecified intracranial hemorrhage |
|  | 433 | Occlusion and stenosis of precerebral arteries |
|  | 434 | Occlusion of cerebral arteries |
|  | 436 | Acute, but ill-defined, cerebrovascular disease |
|  | 437 | Other and ill-defined cerebrovascular disease |
|  | 435 | Transient cerebral ischemia |
|  | V12.54 | Personal history of transient ischemic attack, and cerebral infarction without residual deficits |
| Parkinsonism [2, 4] | 332 | Parkinsonism |
| Encephalopathy [2] | 348.1 | Anoxic brain damage |
|  | 348.3 | Encephalopathy, not elsewhere classified |
| Delirium [5] | 293 | Delirium |
| Polyneuropathy [2] | 356 | Hereditary and idiopathic peripheral neuropathy |
|  | 357 | Inflammatory and toxic neuropathy |
| Mononeuropathy multiplex [2] | 354 | Mononeuritis of upper limb and mononeuritis multiplex |
|  | 355 | Mononeuritis of lower limb |
| Herpes zoster [6] | 053 | Herpes zoster |
| Herpes simplex [7] | 054.3 | Herpetic meningoencephalitis |
|  | 054.4 | With ophthalmic complications |
| Dementia [6] | 290.0 | Senile dementia, uncomplicated |
|  | 290.1 | Presenile dementia |
|  | 290.2 | Senile dementia with delusional or depressive features |
|  | 290.3 | Senile dementia with delirium |
|  | 290.4 | Vascular dementia |
|  | 294.1 | Dementia in conditions classified elsewhere |
|  | 331.0 | Alzheimer's disease |
| Facial nerve disorders [2] | 351 | Facial nerve disorders |
| Nerve roots and plexus disorders [2] | 353 | Nerve roots and plexus disorders |
| Cerebral venous sinus thrombosis [2] | 325 | Cerebral venous sinus thrombosis |
| Neuromuscular junction disorders [2] | 358 | Neuromuscular junction disorders |
| Hypertension [2] | 401-405 | Hypertensive disease |
| Diabetes mellitus [8, 9] | 250 | Diabetes mellitus |
| Coronary heart disease [9, 10, 11] | 410, 412 | Myocardial infarction |
|  | 411, 414 | Other ischemic heart disease |
|  | 413 | Angina pectoris |
|  | V45.81 | Coronary artery bypass graft, neuritis (CABG) |
|  | V45.82 | Percutaneous transluminal coronary angioplasty status (PTCA) |
|  | 428 | Heart failure |
|  | 785.51 | Cardiogenic shock |
|  | 429.2 | Cardiovascular disease, unspecified/other atherosclerotic cardiovascular disease (ASCVD) |
|  | 441 | Aortic aneurysm/dissection |
|  | V12.53 | Personal history of sudden cardiac arrest |
|  | 427.1, 427.3 | Ventricular fibrillation, arrest |
|  | 427.4, 427.5 | Atrial fibrillation, arrest |

Abbreviation: ICD-9-CM: The International Classification of Diseases, Ninth Revision, Clinical Modification

**References:**

1. Harnod T, Lin CL, Kao CH. Epilepsy is associated with higher subsequent mortality risk in patients after stroke: a population-based cohort study in Taiwan. Clinical Epidemiology. 2019;11:247-55. doi: 10.2147/Clep.S201263. PubMed PMID: WOS:000464039500001.

2. Tsai YT, Chen YC, Hsieh CY, Ko WC, Ko NY. Incidence of Neurological Disorders Among HIV-Infected Individuals With Universal Health Care in Taiwan From 2000 to 2010. Jaids-Journal of Acquired Immune Deficiency Syndromes. 2017;75(5):509-16. doi: Doi 10.1097/Qai.0000000000001448. PubMed PMID: WOS:000405556900003.

3. Huang YH, Chi NF, Kuan YC, Chan L, Hu CJ, Chiou HY, et al. Efficacy of phenytoin, valproic acid, carbamazepine and new antiepileptic drugs on control of late-onset post-stroke epilepsy in Taiwan. Eur J Neurol. 2015;22(11):1459-68. Epub 2015/07/07. doi: 10.1111/ene.12766. PubMed PMID: 26148132.

4. Lin PY, Chang SN, Hsiao TH, Huang BT, Lin CH, Yang PC. Association Between Parkinson Disease and Risk of Cancer in Taiwan. Jama Oncology. 2015;1(5):633-40. doi: 10.1001/jamaoncol.2015.1752. PubMed PMID: WOS:000383673100016.

5. Ma IC, Chen KC, Chen WT, Tsai HC, Su CC, Lu RB, et al. Increased Readmission Risk and Healthcare Cost for Delirium Patients without Immediate Hospitalization in the Emergency Department. Clin Psychopharmacol Neurosci. 2018;16(4):398-406. Epub 2018/11/24. doi: 10.9758/cpn.2018.16.4.398. PubMed PMID: 30466212; PubMed Central PMCID: PMCPMC6245300.

6. Chen VC, Wu SI, Huang KY, Yang YH, Kuo TY, Liang HY, et al. Herpes Zoster and Dementia: A Nationwide Population-Based Cohort Study. J Clin Psychiatry. 2018;79(1). Epub 2017/12/16. doi: 10.4088/JCP.16m11312. PubMed PMID: 29244265.

7. Tzeng NS, Chung CH, Lin FH, Chiang CP, Yeh CB, Huang SY, et al. Anti-herpetic Medications and Reduced Risk of Dementia in Patients with Herpes Simplex Virus Infections-a Nationwide, Population-Based Cohort Study in Taiwan. Neurotherapeutics. 2018;15(2):417-29. Epub 2018/03/01. doi: 10.1007/s13311-018-0611-x. PubMed PMID: 29488144; PubMed Central PMCID: PMCPMC5935641.

8. Chang, Y. T., Hwang, J. S., Hung, S. Y., Tsai, M. S., Wu, J. L., Sung, J. M., & Wang, J. D. (2016). Cost-effectiveness of hemodialysis and peritoneal dialysis: A national cohort study with 14 years follow-up and matched for comorbidities and propensity score. Scientific reports, 6, 30266.

9 Lee, Y. C., Lin, C. H., Wu, R. M., Lin, M. S., Lin, J. W., Chang, C. H., & Lai, M. S. (2013). Discontinuation of statin therapy associates with Parkinson disease: a population-based study. Neurology, 81(5), 410-416.

10 Chen IC, Lee CH, Fang CC, et al. Efficacy and safety of ticagrelor versus clopidogrel in acute coronary syndrome in Taiwan: A multicenter retrospective pilot study. J Chin Med Assoc. 2016;79(10):521-530.

11 Lee CH, Cheng CL, Kao Yang YH, Chao TH, Chen JY, Li YH. Cardiovascular and Bleeding Risks in Acute Myocardial Infarction Newly Treated With Ticagrelor vs. Clopidogrel in Taiwan. Circ J. 2018;82(3):747-756.
