# Supplementary material for: Long-term neurological and healthcare burden of adults with Japanese encephalitis: A nationwide study 2000-2015
Source: PLoS Negl Trop Dis. 2021 Sep 14;15(9):e0009703. doi: 10.1371/journal.pntd.0009703 (PMC8486099; doi:10.1371/journal.pntd.0009703)
Supplement: S2 Table — Abbreviations: ICD: International Statistical Classification of Diseases, ICU: intensive care unit, NG: nasogastric tube; PEG: percutaneous endoscopic gastrostomy. (DOCX) [file pntd.0009703.s006.docx]

**S2 Table. ICD procedure codes for the healthcare utilization measured in the present study**

| Healthcare utilization | ICD procedure codes |
| --- | --- |
| ICU stay | 02012A, 02011K, 02013B, 03010E, 03011A, 03011F, 03012B, 03012G, 03013B, 03013H, 03047E, 03048F, 03049G, 03050H |
| Airway suctioning | 47041C, 47042C |
| Ventilator use | 57001B, 57002B, 57023B |
| NG or PEG | 47017C, 47018C, 47018CA |
| Urinary catheterization | 47013C, 47014C |

Abbreviations: ICD: International Statistical Classification of Diseases, ICU: intensive care unit, NG: nasogastric tube; PEG: percutaneous endoscopic gastrostomy.
