# Supplementary material for: Long-term neurological and healthcare burden of adults with Japanese encephalitis: A nationwide study 2000-2015
Source: PLoS Negl Trop Dis. 2021 Sep 14;15(9):e0009703. doi: 10.1371/journal.pntd.0009703 (PMC8486099; doi:10.1371/journal.pntd.0009703)
Supplement: S3 Table — Abbreviations: s.d.: standard deviation, JE: Japanese encephalitis, ER: emergency room. Note: due to small values from the limited sample size at and after the 15th year of JE diagnosis, the cost estimates are not presented in the table. (DOCX) [file pntd.0009703.s007.docx]

**S3 Table. Annual crude healthcare cost breakdown for baseline cost before JE diagnosis, event-year cost at JE diagnosis and annual state/subsequent-year cost after JE diagnosis, and stratified by different medical services (i.e., inpatient, outpatient, emergency room, pharmacy and rehabilitation-related) among a total of 352 study patients (in 2019 USD)**

|  | **n** | **mean** | **s.d.** | **median** | **max.** | **min.** | **Q1** | **Q3** |
| --- | --- | --- | --- | --- | --- | --- | --- | --- |
| **Baseline cost** | 352 | 1,118.77 | 3,328.37 | 223.26 | 31,763.17 | 0 | 53.96 | 718.82 |
| Inpatient |  | 354.89 | 2,013.44 | 0 | 31,215.80 | 0 | 0 | 0 |
| Outpatient |  | 673.85 | 2,264.19 | 178.06 | 21,138.62 | 0 | 39.65 | 485.11 |
| ER |  | 30.90 | 129.23 | 0 | 1,415.52 | 0 | 0 | 0 |
| Pharmacy |  | 26.39 | 85.11 | 0 | 718.54 | 0 | 0 | 9.58 |
| Rehabilitation-related |  | 32.57 | 309.81 | 0 | 5,103.94 | 0 | 0 | 0 |
| **Event-year cost** | 352 | 19,628.99 | 45,360.55 | 7,376.55 | 422,845.82 | 80.06 | 3,643.29 | 18,165.57 |
| Inpatient |  | 11,749.06 | 40,815.11 | 2,620.32 | 399,198.92 | 0 | 606.14 | 6,228.72 |
| Outpatient |  | 1,037.64 | 2,542.61 | 466.40 | 25,151.15 | 0 | 124.99 | 1,072.80 |
| ER |  | 458.47 | 421.24 | 391.26 | 2,215.73 | 0 | 106.80 | 664.43 |
| Pharmacy |  | 36.12 | 144.88 | 0 | 2,012.70 | 0 | 0 | 13.84 |
| Rehabilitation-related |  | 259.41 | 983.19 | 0 | 10,058.41 | 0 | 0 | 0 |
| **Subsequent-year cost** |  | 0 | 0 | 0 | 0 | 0 | 0 | 0 |
| **2^nd^ year** | 305 | 3,262.92 | 11,308.67 | 480.78 | 105,868.06 | 0 | 105.64 | 1,523.71 |
| Inpatient |  | 1,811.94 | 10,527.44 | 0 | 105,085.02 | 0 | 0 | 0 |
| Outpatient |  | 924.23 | 2,453.23 | 314.32 | 22,900.72 | 0 | 82.64 | 840.65 |
| ER |  | 68.68 | 229.91 | 0 | 2,051.56 | 0 | 0 | 20.62 |
| Pharmacy |  | 43.53 | 137.96 | 0 | 934.43 | 0 | 0 | 11.15 |
| Rehabilitation-related |  | 160.17 | 786.35 | 0 | 7,382.72 | 0 | 0 | 0 |
| **3^rd^ year** | 280 | 3,596.36 | 15,114.05 | 381.19 | 156,612.37 | 0 | 88.88 | 1,160.43 |
| Inpatient |  | 2,450.99 | 14,546.11 | 0 | 156,579.66 | 0 | 0 | 0 |
| Outpatient |  | 799.82 | 2,538.07 | 246.58 | 24,671.73 | 0 | 59.06 | 613.47 |
| ER |  | 64.79 | 254.44 | 0 | 3,051.28 | 0 | 0 | 3.12 |
| Pharmacy |  | 48.34 | 172.86 | 0 | 1,603.29 | 0 | 0 | 12.68 |
| Rehabilitation-related |  | 98.88 | 518.18 | 0 | 5,185.74 | 0 | 0 | 0 |
| **4^th^ year** | 253 | 2,444.64 | 7,839.14 | 346.41 | 69,738.60 | 0 | 89.68 | 1,113.02 |
| Inpatient |  | 1,435.34 | 7,181.82 | 0 | 68,211.28 | 0 | 0 | 0 |
| Outpatient |  | 804.61 | 2,383.45 | 229.78 | 22,048.27 | 0 | 69.60 | 656.56 |
| ER |  | 43.59 | 140.20 | 0 | 1,634.91 | 0 | 0 | 0 |
| Pharmacy |  | 49.08 | 160.81 | 2.68 | 1,596.54 | 0 | 0 | 15.53 |
| Rehabilitation-related |  | 86.50 | 543.17 | 0 | 5,202.07 | 0 | 0 | 0 |
| **5^th^ year** | 220 | 2,650.65 | 15,248.38 | 406.60 | 219,833.81 | 0 | 91.19 | 960.12 |
| Inpatient |  | 1,509.07 | 14,923.31 | 0 | 219,289.55 | 0 | 0 | 0 |
| Outpatient |  | 764.25 | 1,942.53 | 282.95 | 21,572.31 | 0 | 68.97 | 682.58 |
| ER |  | 38.32 | 124.70 | 0 | 925.45 | 0 | 0 | 0 |
| Pharmacy |  | 36.57 | 104.39 | 2.63 | 664.76 | 0 | 0 | 16.65 |
| Rehabilitation-related |  | 90.39 | 569.55 | 0 | 5,468.70 | 0 | 0 | 0 |
| **6^th^ year** | 197 | 1,722.40 | 4,581.70 | 297.32 | 33,322.60 | 0 | 85.88 | 854.76 |
| Inpatient |  | 769.42 | 3,375.24 | 0 | 28,316.28 | 0 | 0 | 0 |
| Outpatient |  | 783.32 | 2,648.25 | 217.15 | 28,346.09 | 0 | 71.78 | 615.41 |
| ER |  | 40.62 | 122.97 | 0 | 968.37 | 0 | 0 | 0 |
| Pharmacy |  | 23.09 | 76.11 | 2.79 | 809.89 | 0 | 0 | 16.11 |
| Rehabilitation-related |  | 52.46 | 381.52 | 0 | 4,604.68 | 0 | 0 | 0 |
| **7^th^ year** | 174 | 2,427.30 | 9,128.87 | 397.42 | 86,611.58 | 0 | 125.50 | 1,131.35 |
| Inpatient |  | 1,322.68 | 8,466.22 | 0 | 86,611.58 | 0 | 0 | 0 |
| Outpatient |  | 908.14 | 2,595.11 | 268.81 | 21,943.20 | 0 | 66.70 | 680.30 |
| ER |  | 42.45 | 135.82 | 0 | 1,198.88 | 0 | 0 | 0 |
| Pharmacy |  | 36.92 | 102.93 | 0 | 729.49 | 0 | 0 | 21.85 |
| Rehabilitation-related |  | 52.04 | 396.51 | 0 | 4,342.22 | 0 | 0 | 0 |
| **8^th^ year** | 157 | 1,812.78 | 5,796.59 | 394.89 | 43,512.94 | 0 | 100.72 | 931.51 |
| Inpatient |  | 593.11 | 3,790.65 | 0 | 42,089.23 | 0 | 0 | 0 |
| Outpatient |  | 1,103.99 | 3,697.97 | 293.90 | 29,019.94 | 0 | 77.70 | 649.44 |
| ER |  | 39.33 | 177.41 | 0 | 1,860.71 | 0 | 0 | 0 |
| Pharmacy |  | 36.10 | 91.67 | 3.02 | 532.90 | 0 | 0 | 18.80 |
| Rehabilitation-related |  | 40.25 | 336.21 | 0 | 3,915.57 | 0 | 0 | 0 |
| **9^th^ year** | 144 | 1,266.22 | 3,301.70 | 318.06 | 29,344.40 | 0 | 106.20 | 882.26 |
| Inpatient |  | 147.11 | 790.05 | 0 | 7,316.49 | 0 | 0 | 0 |
| Outpatient |  | 993.31 | 2,985.68 | 274.22 | 26,050.49 | 0 | 77.49 | 618.40 |
| ER |  | 50.80 | 297.54 | 0 | 3,293.91 | 0 | 0 | 0 |
| Pharmacy |  | 42.72 | 129.05 | 3.28 | 1,053.96 | 0 | 0 | 21.72 |
| Rehabilitation-related |  | 32.29 | 231.95 | 0 | 2,136.81 | 0 | 0 | 0 |
| **10^th^ year** | 119 | 1,627.86 | 4,463.84 | 418.11 | 35,277.45 | 0 | 123.21 | 1,126.02 |
| Inpatient |  | 614.75 | 3,348.57 | 0 | 34,515.96 | 0 | 0 | 0 |
| Outpatient |  | 875.89 | 2,888.58 | 279.25 | 23,381.96 | 0 | 79.30 | 745.16 |
| ER |  | 40.97 | 119.62 | 0 | 761.49 | 0 | 0 | 0 |
| Pharmacy |  | 45.38 | 110.78 | 3.72 | 676.60 | 0 | 0 | 25.12 |
| Rehabilitation-related |  | 37.41 | 223.54 | 0 | 1,841.23 | 0 | 0 | 0 |
| **11^th^ year** | 101 | 1,101.14 | 3,258.25 | 326.59 | 25,459.93 | 0 | 104.89 | 862.26 |
| Inpatient |  | 58.93 | 318.41 | 0 | 2,890.53 | 0 | 0 | 0 |
| Outpatient |  | 915.75 | 3,149.32 | 253.03 | 24,485.72 | 0 | 77.28 | 626.89 |
| ER |  | 40.22 | 132.46 | 0 | 970.45 | 0 | 0 | 0 |
| Pharmacy |  | 48.90 | 115.74 | 6.18 | 747.67 | 0 | 0 | 41.38 |
| Rehabilitation-related |  | 37.33 | 222.44 | 0 | 1,696.76 | 0 | 0 | 0 |
| **12^th^ year** | 78 | 1,382.38 | 5,157.36 | 264.15 | 44,436.51 | 0 | 106.20 | 962.46 |
| Inpatient |  | 228.69 | 1,119.18 | 0 | 9,418.79 | 0 | 0 | 0 |
| Outpatient |  | 745.43 | 2,123.59 | 189.80 | 15,446.60 | 0 | 84.87 | 438.63 |
| ER |  | 24.66 | 88.89 | 0 | 504.89 | 0 | 0 | 0 |
| Pharmacy |  | 42.47 | 106.69 | 3.79 | 683.94 | 0 | 0 | 27.37 |
| Rehabilitation-related |  | 34.13 | 153.56 | 0 | 998.03 | 0 | 0 | 0 |
| **13^th^ year** | 56 | 1,390.64 | 3,868.15 | 205.27 | 21,769.13 | 0 | 82.86 | 801.71 |
| Inpatient |  | 294.52 | 1,725.12 | 0 | 12,791.24 | 0 | 0 | 0 |
| Outpatient |  | 1,001.22 | 3,418.48 | 168.31 | 21,691.33 | 0 | 65.02 | 555.94 |
| ER |  | 25.99 | 98.85 | 0 | 613.53 | 0 | 0 | 0 |
| Pharmacy |  | 28.85 | 91.98 | 3.69 | 617.15 | 0 | 0 | 14.03 |
| Rehabilitation-related |  | 40.06 | 185.58 | 0 | 1,300.68 | 0 | 0 | 0 |
| **14^th^ year** | 38 | 1,458.82 | 3,138.00 | 323.49 | 12,912.92 | 0 | 69.10 | 931.29 |
| Inpatient |  | 458.24 | 1,944.55 | 0 | 11,689.58 | 0 | 0 | 0 |
| Outpatient |  | 891.54 | 2,176.47 | 260.61 | 10,730.47 | 0 | 48.83 | 547.07 |
| ER |  | 22.98 | 103.91 | 0 | 630.44 | 0 | 0 | 0 |
| Pharmacy |  | 35.93 | 89.55 | 3.83 | 477.27 | 0 | 0 | 15.73 |
| Rehabilitation-related |  | 50.12 | 244.30 | 0 | 1,481.05 | 0 | 0 | 0 |

Abbreviations: s.d.: standard deviation, JE: Japanese encephalitis, ER: emergency room.

Note: due to small values from the limited sample size at and after the 15^th^ year of JE diagnosis, the cost estimates are not presented in the table.
