# Supplementary material for: Long-term neurological and healthcare burden of adults with Japanese encephalitis: A nationwide study 2000-2015
Source: PLoS Negl Trop Dis. 2021 Sep 14;15(9):e0009703. doi: 10.1371/journal.pntd.0009703 (PMC8486099; doi:10.1371/journal.pntd.0009703)
Supplement: S4 Table — Abbreviation: JE: Japanese encephalitis. aNeurological disease history included polyneuropathy/mononeuropathy multiplex and nerve root and plexus disorders. (DOCX) [file pntd.0009703.s008.docx]

**S4 Table. Results of univariate Cox proportional hazards model for all-cause death as study outcome**

|  | **Hazard ratio** | **95% lower confidence limit** | **95% upper confidence limit** |
| --- | --- | --- | --- |
| **Age at JE diagnosis (ref: age 20-40 years)** | | | |
| 40-64 years | 2.172 | 1.192 | 3.957 |
| $\geq$65 years | 5.340 | 2.439 | 11.690 |
| **Gender (ref: male)** | | | |
| Female | 0.837 | 0.500 | 1.402 |
| **Diagnosis year (ref: 2000-2004)** | | | |
| 2005-2009 | 1.212 | 0.682 | 2.155 |
| 2010-2014 | 0.998 | 0.503 | 1.980 |
| **JE vaccination history (ref: no vaccine)** | | | |
| Born in 1963-1969: 2 doses | 0.307 | 0.139 | 0.680 |
| Born in 1970-1975: 3 doses | 0.457 | 0.206 | 1.011 |
| Born after 1976: 4 doses | 0.162 | 0.050 | 0.520 |
| **Medical history (ref: none)** |  |  |  |
| Comorbid diabetes | 4.732 | 2.701 | 8.290 |
| Comorbid hypertension | 2.980 | 1.776 | 5.001 |
| Comorbid coronary heart disease | 3.491 | 1.660 | 7.344 |
| **Incident events (ref: none)** |  |  |  |
| Epilepsy/convulsions | 2.129 | 1.255 | 3.613 |
| Stroke | 1.667 | 0.975 | 2.852 |
| Parkinsonism | 0.322 | 0.101 | 1.027 |
| Encephalopathy/delirium | 1.174 | 0.598 | 2.305 |
| Neurological events^a^ | 0.713 | 0.425 | 1.194 |

Abbreviation: JE: Japanese encephalitis.

^a^Neurological events included polyneuropathy/mononeuropathy multiplex and nerve root and plexus disorders.
